# Measurement of public health benefits of physical activity: validity and reliability study of the international physical activity questionnaire in Hungary

**DOI:** 10.1186/s12889-020-08508-9

**Published:** 2020-08-17

**Authors:** Pongrác Ács, József Betlehem, András Oláh, Józef Bergier, Csaba Melczer, Viktória Prémusz, Alexandra Makai

**Affiliations:** 1 Faculty of Health Sciences, University of Pécs, Pécs, Hungary; 2 Pope John Paul II. State School of Higher Education, Biała Podlaska, Poland

**Keywords:** Metabolic equivalent, Physical activity, Questionnaire, Accelerometer, Reliability, Validity, IPAQ

## Abstract

**Background:**

To ensure accurate measurement of the health benefits of habitual physical activity in large sample epidemiological studies, physical activity questionnaires (PAQs) are the most feasible methods. Therefore, the purpose of this study was the validation and cultural adaptation to the Hungarian population of the International Physical Activity Questionnaire (IPAQ-HL).

**Methods:**

A cross-sectional study among Hungarian healthy adults (age 21.375 ± 1.940 years, *n* = 120) was performed comparing measures of last 7 days IPAQ-HL self- administered questionnaire and obtained accelerometer (Actigraph GT3X) data for concurrent validity, reassessed by a random subsample (*n* = 33) to measure reliability.

**Results:**

Our results indicate acceptable criterion validity for total physical activity, moderate to vigorous physical activity (*R* = 0.387, *p* < 0.001; *R* = 0.331 *p* < 0.001 respectively) and moderate physical activity (*R* = 0.193, *p* = 0.034). The ICC scores revealed moderate to good correlations (ICC = 0.744–0.942, *p* < 0.001). Moderate Kaiser–Meyer–Olkin measure (0.531, *p* < 0.001) and good reproducibility for vigorous, moderate to vigorous and moderate activities was found for IPAQ-HL in the studied population. Nevertheless, like analogous self-reports in other languages, it overestimates the time spent on physical activity.

**Conclusions:**

IPAQ-HL proved to be a reasonably valid measure for population prevalence epidemiological studies and is suggested for use to develop public health policy recommendations or to optimize public health interventions. However, the results on vigorous activity should be interpreted with caution, the questionnaire showed moderate validity for this particular intensity.

## Background

It is generally accepted that physical activity beyond improving fitness and endurance levels plays a significant role in the promotion of physical and psychological health by reducing chronic diseases (cardiovascular and metabolic disorders, diabetes, cancer, obesity and osteoporosis), mental disorders (chronic stress, anxiety and depression) and decreasing all causes of mortality [1–6].

For the public health benefits mentioned above, implementation of the EU recommendation on health-enhancing physical activity (HEPA) in the countries of the European Union (EU) is a burning issue [7].

For obtaining the health benefits of PA the World Health Organization (WHO) also proposes minimum activity levels: at least 150 min of moderate- or 75 min of vigorous physical activity (PA) on a weekly basis for the general population aged 18 to 64 years [8]. Following the definition of WHO, in the current study PA was also specified not only as regularly practiced exercises but also as exercise that includes all types of bodily movement produced by skeletal muscles that requires energy expenditure [9].

Physical activity questionnaires (PAQs) are the most feasible methods [10] to ensure accurate measurement of habitual physical activity in large sample epidemiological studies on the relationship between physical activity and health, on the fulfilment of recommendations or even on seeking an appropriate pattern of physical activity for maintaining health benefits.

To obtain internationally comparable data on health–related physical activity that people engage in as part of their everyday lives, the International Physical Activity Questionnaire (IPAQ) is one of the recommended and - for the general population - the most widely used PAQs [11, 12]. The questionnaire is validated in 7-item short and 27-item long form, with a reference period of the last 7 days. The IPAQ long form (IPAQ-L) queries 5 activity domains independently and provides specific details on PA intensity levels and differentiates between usual week days and weekend days by measuring sitting time. For PA assessment IPAQ-L is used worldwide [13], as well as in the Central and Eastern European (CEE) countries and in Hungary [14–16]. However, no validated Hungarian version of this questionnaire exists to this date.

Therefore, the purpose of this study was the validation and cultural adaptation to the Hungarian population of the IPAQ-L. We aimed to develop a Hungarian version of the IPAQ long form (IPAQ-HL) - equivalent to the original English version, culturally adapt it to the target population and assess its validity and reproducibility on a sample of healthy young adults in Hungary.

## Methods

### Study design

A cross-sectional study was performed comparing measures of last 7 days of PA by the Hungarian long version of IPAQ self- administered questionnaire and obtained accelerometer (Actigraph GT3X) data for concurrent validity. To measure the reliability of the IPAQ-HL questionnaire, the results were reassessed by a random subsample.

### Sample

Three hundred students with convenience sampling were recruited from the University of Pécs (Pécs, Hungary), from different faculties (Faculty of Medicine *N* = 90, Technology and Informatics *N* = 88, and Health *N* = 122) from January 2018 to July of 2018. The interested students were contacted by e-mail. Eligibility criteria for inclusion were be able to speak and understand Hungarian, good health status, being a student at the University of Pécs. Exclusion criteria for study were physical inability or illness, which would be restrict the performance of normal lifestyle activities. Any participant met the previously determined exclusion criterions.

**Fig. 1:**
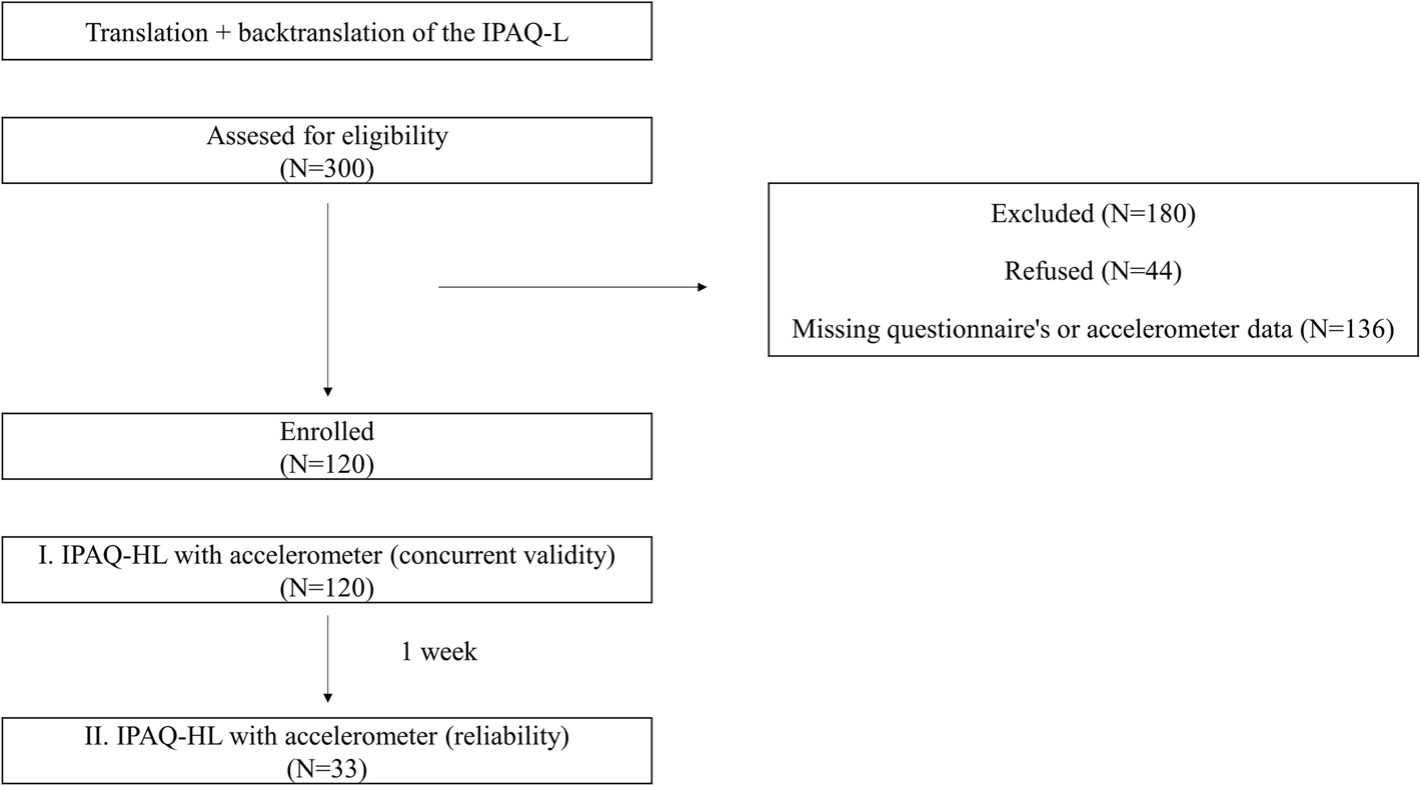
Participant flow diagram for a validation study of the Hungarian version of the International Physical Activity Questionnaires long form (IPAQ-HL)

Finally, 44 students refused participation, and 136 were classified as not eligible due to missing questionnaire or accelerometer data. The final sample contained 120 students (male *N* = 56, female *N* = 64) resulting in a response rate of 40%. The participant flow and exclusions are presented in Fig. 1.

The study protocol and the instructions to wear the ActiGraph were explained by trained interviewers (researchers). After being informed of the study aims, the students provided informed consent. Then, they completed the IPAQ-HL and the socio-demographic and lifestyle questionnaire, including age, educational level, income, marital status, and lifestyle habits. In our research we used the self-administered version of the questionnaire, added trained researchers to support the participants and to register anthropometric and body composition data at baseline and to explain how to wear the accelerometers and initialize the devices. To minimalize the inter-rater bias all researcher received training before using the accelerometers and starting the data collection. After wearing time researchers recalled the participants to assemble the accelerometers.

### Measurements

#### IPAQ

Students self-reported physical activity through the long version of the International Physical Activity Questionnaire [13]. We used the method for cross-cultural adaptation recommended by IPAQ committee [17] which proposes the forward translation of the IPAQ followed by a back translation into English by two independent experts. A few cultural adaptations were made to the original items to reflect the reality in Hungary, like the replacement of the English word vigorous, which was hardly understood in Hungarian, with the word” intense”. Then, the translated questionnaire was pilot tested in a convenience sample of 10 persons. The draft items should not be revised upon reviewing the justifying results of the preliminary pilot testing. The final questionnaire was assessed on the 0th day then it was reassessed in a random sample (*n* = 33) of the participants with a seven-day recall [18].

The IPAQ-HL covers 5 domains (work, transportation, domestic and recreational activities and sitting time) with a time frame of the last 7 days and records how many days, and in a day how many hours and minutes (with a minimum length of 10 min) were spent with a kind of moderate or vigorous activity.

In our study an activity type specific truncation rule with a maximum of PA was applied. As suggested in the scoring protocol guidelines, more than ‘3 h’ or ‘180 min’ were re-coded to ‘180 min’ in a new variable.

The total moderate to vigorous physical activity (MVPA) was calculated [17]. We calculated for each item the metabolic equivalent of task (MET) minute values according to the scoring protocol (walking 3.3 METs, moderate 4 METs, vigorous 8 METs, cycling 6 METs, outdoor vigorous domestic activities 5.5 METs and inside domestic activities 3 METs). Consensually, one MET was evaluated to be equal to energy expenditure during rest and interpreted approximately as equal to 3.5 ml O2/kg min in healthy adults [19].

Additionally to IPAQ-HL, socioeconomic status was assessed by gender, age, education, employment status, marital status and household inventory.

#### ActiGraph

A triaxial accelerometer (ActiGraph GTX3+, ActiGraph, Pensacola, FL) was used to assess PA. The device was initialized to collect data at a sample rate of 30 Hz and 60 s epochs and normal filter option. Participants were advised to wear the accelerometer on the right hip for seven consecutive days during waking hours excluding contact sports, washing, bathing, swimming or sleeping activities. A minimum of 5–7 days of valid wear time (480 min of wear time per day) was required for inclusion into the analysis [20]. Non-wear time was defined as 60 or more minutes of zeros. Analysis of the accelerometer data was conducted using ActiLife 6 software to initialize the accelerometer and to download results, raw data was converted with Freedson cut points [21]. Average daily time in moderate to vigorous physical activity (MVPA) (min/day) and sedentary behaviour (SB) (min/day) were calculated [22, 23].

#### Anthropometric data

Anthropometric measurements such as height (m) and weight (kg) were measured at baseline using a portable wall mounted stature meter and Omron B510 (OMRON Healthcare Europe BV, Hoofddorp NL), while the students were dressed in light clothing without shoes. The height was measured and recorded to the nearest 0.5 cm, in standing position, without shoes, when the shoulders were in normal position. Body mass index (BMI; kg/m2) data were calculated by Omron B510 as weight (kg) divided by height (m) squared and classified according to the WHO [24]. Then, body fat, muscle and visceral fat were also registered using Omron B510. Waist and hip circumference (cm) were measured by anthropometric tape measure.

#### Validation process

In order to allow for comparisons with other validity studies, the Edinburgh Framework for validity and reliability [25], as well as the COSMIN checklist [26, 27] were applicable for the analysis.

### Statistical analysis

To present the quantitative data mean (standard deviation, SD) and median (inter quartile range, IQR) were computed. Normality of the data was tested using Kolmogorov-Smirnov test (where the data was considered normally distributed *p* > 0.05). Mann-Whitney U test was calculated to measure the gender differences in PA levels. Factor analysis was conducted using principal component analysis (PCA) and varimax rotation. The Kaiser–Meyer–Olkin (KMO) was calculated along with Bartlett’s test and anti–image correlation. We assessed the construct validity of the IPAQ-HL questionnaire using Spearman’s rank correlation coefficient between the ActiGraph and IPAQ (correlation coefficients were considered as > 0.400 good validity, 0.300–0.400 moderate validity, < 0.300 poor validity [28]. We performed Bland-Altman analyses to evaluate the extent of agreement between the accelerometer and the IPAQ. To measure the internal consistency reliability Cronbach Alpha was calculated. Intraclass correlation coefficient (ICC) was used for test retest reliability analysis of the IPAQ where above 0.750 means good reliability, 0.500–0.750 moderate reliability and lower means poor reliability [29]. The statistical analysis were performed using IBM SPSS 22.0, confidence interval of 95% was used, and *p* value of < 0.05 was considered statistically significant.

## Results

### Descriptive results

In total, 120 students remained in the study, general characteristics (age 21.375 years [SD 1.940].), BMI and body composition sample were shown in Table 1.

**Table 1:** Sample characteristics (*n* = 120)

	TOTAL		MALE		FEMALE		P^a^
N	120		56		64		
	Mean	SD	Mean	SD	Mean	SD	
Age (Years)	21.529	1.751	21.709	1.941	21.375	1.569	0.470
BMI (kg/m^2^)	23.746	3.813	23.994	2.692	23.527	4.594	0.158
Body fat (%)	27.209	12.054	21.864	14.246	31.930	6.971	< 0.001*
Muscle (%)	34.004	6.650	39.943	4.244	28.758	2.899	< 0.001*
Visceral fat (%)	4.770	2.431	5.623	2.513	4.017	2.103	< 0.001*
Hip circumference (cm)	100.159	9.089	102.000	7.925	98.533	9.785	0.043*
Waist circumference (cm)	78.816	13.404	85.151	8.254	73.311	14.597	< 0.001*
Exercise regularly (n, %)^b^	63	52.500	39	69.643	24	37.500	0.071
Good/Very good SRH (n, %)^c^	81	67.500	39	69.643	42	65.625	0.393

*Sample characteristics in the validation study of the Hungarian version of the International Physical Activity Questionnaires long form (IPAQ-HL).*

^*a*^presents mean intergroup differences between the genders, *p < 0.05

^*b*^*self-report sporting activity at least three times weekly*

^*c*^ *good or very good self-rated health*

Gender differences were found in body fat, muscle, visceral fat, waist and hip circumference. However, the BMI was considered normal (< 25) in cases of female and male students as well, and we did not find significant differences in BMI values by gender.

In the physical activity measurement, we found significant differences between genders according to the ActiGraph vigorous (*p* = 0.048) and sitting time (*p* = 0.018) and in IPAQ-HL data according to MVPA MET min / week (*p* = 0.048), TOTAL MET min / week (*p* = 0.044), leisure time MET min / week (*p* = 0.017) and vigorous MET min / week (*p* = 0.017). The female participants spent less time with vigorous activities according to the accelerometer and IPAQ results than the males. The work, transportation, domestic physical activity level and sitting time of the students were not significantly different according to gender.

Comparing the accelerometer and IPAQ-HL data we found, in general, higher values of PA with IPAQ than with ActiGraphs. Respondents reported 10.117 (±13.080)-times higher vigorous activity in IPAQ-HL questionnaire compared to the accelerometer results. The moderate activity was 1.470 (±3.813) times more, the MVPA 2.646 (±8.898) times more with IPAQ-HL compared to accelerometers. However, accelerometer-measured sitting time was 4.357 (±3.759)-times more than the IPAQ-HL results. The domestic domain was the lowest of different activities by IPAQ-HL result, 626.098 (±1003.992) MET min. The recreational domain was the highest, 2552.736 (±2388.239) (Table 2).

**Table 2:** Physical activity levels based on IPAQ-HL and ActiGraph GT3X results with respect to genders (*n* = 120)

	PA by intensity or domain	Total	Male	Female	p^a^
		Mean (SD)	Mean (SD)	Mean (SD)	
ActiGraph GT3X	total MVPA (min/week)	345.518 (139.050)	342.708 (164.060)	347.977 (114.063)	0.873
	vigorous (min/week)	6.533 (18.485)	9.527 (22.607)	3.914 (13.577)	0.048*
	moderate (min/week)	338.574 (136.611)	332.938 (158.114)	343.505 (115.642)	0.784
	sedentary (min/week)	9106.751 (370.722)	9037.685 (437.122)	9167.185 (291.267)	0.018*
IPAQ-HL	total MVPA (min/week)	603.090 (494.402)	647.392 (509.743)	567.362 (482.866)	*0.364*
	total moderate (min/week)	348.950330.597)	328.054 (316.808)	367.186 (343.652)	0.497
	total vigorous (min/week)	257.143 (274.482)	325.672 (298.140)	201.879 (242.354)	0.017*
	sitting time (min/week)	2593.650 (1052.028)	2618.750 (1099.952)	2571.688 (1016.487)	0.666
	total MVPA (MET)	4166.250 (3676.714)	4888.426 (3987.062)	3538.786 (3289.567)	0.048*
	total moderate (MET)	1417.939 (1233.464)	1507.933 (1383.353)	1337.588 (1088.906)	0.805
	total vigorous (MET)	2057.146 (2195.859)	2605.373 (2385.115)	1615.028 (1938.914)	0.017*
	total (MET)	3365.161 (2853.493)	3956.239 (3027.332)	2860.058 (2618.856)	0.044*
	total work (MET)	2219.115 (3351.094)	2681.508 (4041.539)	1808.928 (2555.712)	0.964
	total transportation (MET)	1755.993 (1393.393)	1813.025 (1480.571)	1706.440 (1323.402)	0.800
	total domestic (MET)	626.098 (1003.992)	620.545 (1006.902)	630.844 (1009.696)	0.551
	total recreational (MET)	2552.736 (2388.239)	2914.398 (2911.593)	2238.505 (1782.962)	0.017*
	total walking (MET)	2018.479 (2024.153)	2586.557 (2424.213)	1540.573 (1470.630)	0.725

*Physical activity levels described by intensity or domain in the validation study of the Hungarian version of the International Physical Activity Questionnaires long form (IPAQ-HL),* ^*a*^presents mean intergroup differences between the genders, *p < 0.05.

### Concurrent validity

We tested the concurrent validity of the IPAQ-HL – ActiGraph GT3X results using Spearman’s rank correlation. Moderate significant correlation was found between total and MVPA results of IPAQ-HL and accelerometers (*R* = 0.387, *p* < 0.001; *R* = 0.331 *p* < 0.001 respectively).

There was no significant correlation between vigorous intensity activities, but the vigorous activity of IPAQ-HL showed significant correlation with moderate accelerometer data (*R* = 0.507, *p* < 0.001) and total MVPA accelerometer data (*R* = 0.483, *p* < 0.001). Furthermore, the moderate intensity ActiGraph data significantly correlated with IPAQ-HL moderate (*R* = 0.193, *p* < 0.034).

### Content validity

Factor analysis (Principal component analysis (PCA) was performed using all items of IPAQ-HL (all have adequate communality with other items) with varimax rotation. A moderate KMO measure was found (0.531) with significant Bartlett’s test of Sphericity (*p* < 0.001). The total variance explained 63.615%. The factor 1 was work with the vigorous, moderate, walking during work items, furthermore the walking during transportation was also found in factor 1 (17.628%). Domestic activities were found in factor 2 (the outdoor vigorous, outdoor moderate, indoor moderate) which explained the 15.558% of variance. Additionally, the factor 3 was cycling for travelling which item explained the 10.271% of variance. The factor 4 was the leisure time activity factor with vigorous, moderate, walking in leisure time items explaining the 10.247% of the variance. The last factor was sitting time explaining the 9.912% of variance.

### Internal consistency reliability

The IPAQ-HL domain items showed moderate agreement, in the work domain Cronbach’s Alpha (CA) was 0.695 (ICC 0.604–0.771), in transportation domain CA was 0.671 (ICC 0.571–0.755), in the domestic PA item’s CA was 0.728 (ICC 0.646–0.797) and in the recreational activity items CA was 0.458 (ICC: 0.297–0.595). Almost high agreement was found for total IPAQ-HL item’s (CA 0.720, ICC 0.642–0.789).

### Test-retest reliability

Seven days after the first measurement some of the participants completed the IPAQ-HL again, recalling their physical activity during the week when they wore the accelerometers. Thirty-three students participated in this test-retest analysis; Table 3 lists the intraclass correlation coefficient (ICC) for the test-retest reliability and 95% CI for each scale of the questionnaire. Overall score and domains showed acceptable correlation coefficients. Furthermore, the Spearman’s rank correlation showed work, transportation, domestic, leisure time R and p.

**Table 3:** Test-retest reliability of the IPAQ-HL in Hungarian sample (*n* = 33)

IPAQ-HL by intensity or domain	Spearman’s rho		ICC (lower-upper)
total (MET)			
total work (MET)	R	0.765	0.942
	p	< 0.001^**^	(0.849–0.978)
total transportation (MET)	R	0.802	0.894
	p	< 0.001^**^	(0.733–0.958)
total domestic (MET)	R	0.939	0.927
	p	< 0.001^**^	(0.810–0.972)
total recreational (MET)	R	0.931	0.859
	p	< 0.001^**^	(0.645–0.944)
total walking (MET)	R	0.878	0.744
	p	< 0.001^**^	(0.354–0.899)
total moderate (MET)	R	0.778	0.863
	p	< 0.001^**^	(0.645–0.947)
total vigorous (MET)	R	0.985	0.932
	p	< 0.001^**^	(0.824–0.974)
sitting time (min/week)	R	0.841	0.912
	p	< 0.001^**^	(0.778–0.965)

Test-retest reliability described by intensity or domain in the validation study of the Hungarian version of the International Physical Activity Questionnaires long form (IPAQ-HL), *p < 0.05, **p < 0.01.

### Bland Altman plots

**Fig. 2:**
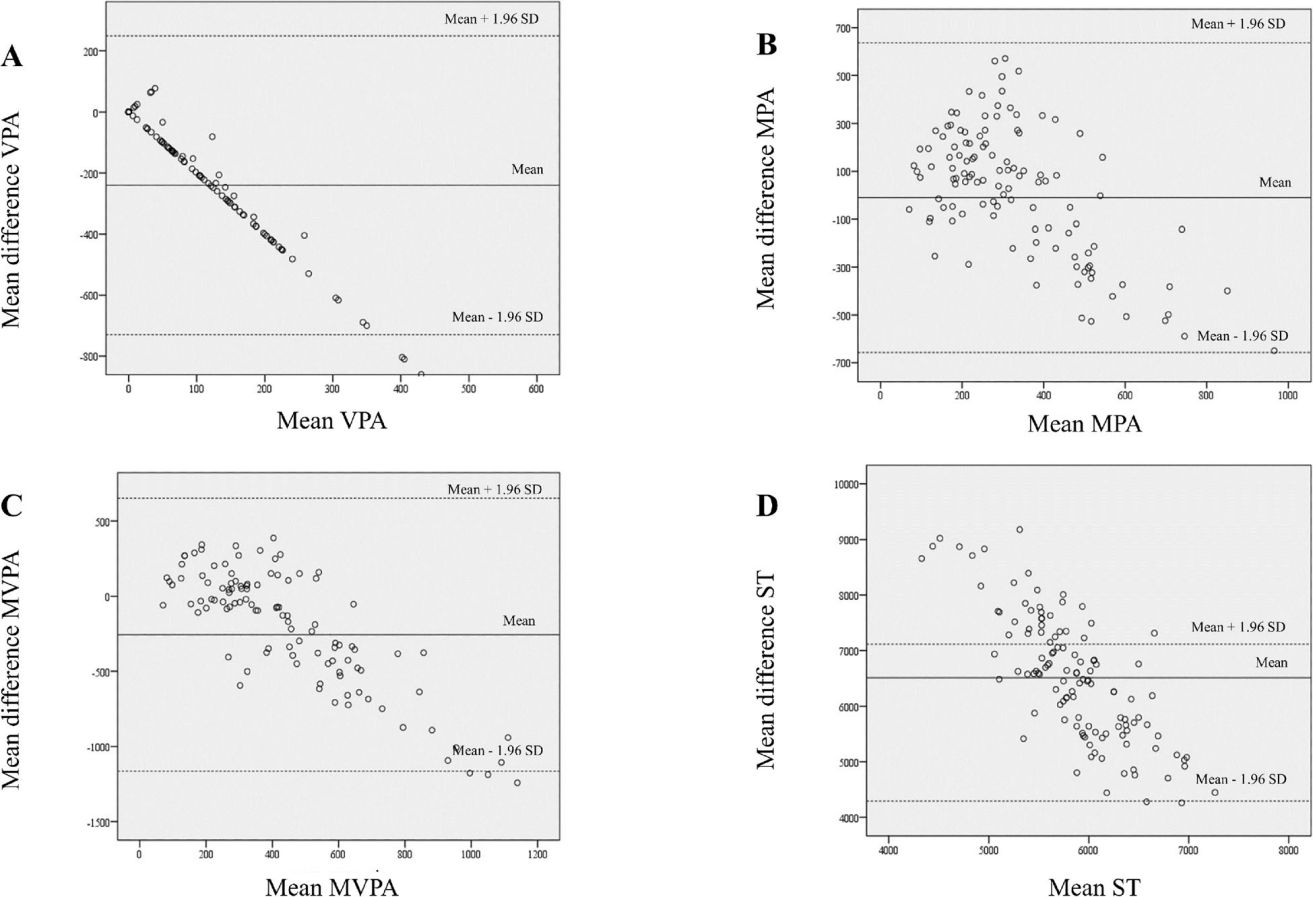
Bland Altman plots of the IPAQ-HL in Hungarian sample (*n* = 120). Bland-Altman plots for the agreement of data assessed with ActiGraph GT3X accelerometer and with the IPAQ-HL in the validation study of the Hungarian version of the International Physical Activity Questionnaires long form. MVPA, moderate-to-vigorous physical activity (min/week); MPA, moderate physical activity (min/week); VPA, vigorous physical activity (min/week); ST, sitting time (min/week). The metric for both x- and y-axes in each graph (**a**-**d**) are the z-scores for mean (mean of the Actigraph GT3X accelerometer and IPAQ-HL scores) domain scores and the difference (difference of the Actigraph GT3X accelerometer and IPAQ-HL scores) between scores, _______ = observed average agreement; _ _ _ _ _ _ =95% limit of agreement; y=0 line of perfect average agreement

Figure 2 Bland Altman plots illustrate the agreement in the values of total MVPA (Mean difference = (−255.838) min/week, 95% limit of agreement = 652.331- (− 1164.010)), vigorous (Mean difference = (− 240.475) min/week, 95% limit of agreement = 248.506- (− 729.456), moderate physical activity (Mean difference = (−10.351) min/week, 95% limit of agreement = 636.3946 – (− 657.097), and sitting time (Mean difference = 6513.101 min/week, 95% limit of agreement = 8733.011–4293.192).

## Discussion

As a subjective measurement tool, the Hungarian version of the International Physical Activity Questionnaire - Long (IPAQ-HL) was used to self-report everyday health-enhancing physical activity and it has acceptable validity for the measurement of total PA, MVPA and moderate PA and good reliability coefficients for application in the population studied. However, the results on vigorous activity should be interpreted with caution, the questionnaire showed moderate validity for this particular intensity.

In the 12-country reliability and validity study of Craig et al. respondents self-reported the median of 3699 MET-min weekly in total with IPAQ. We found similar total MET result in mean 3365.161 (2853.493), but median was lower, 2743.700 (1351.300–4700.250) [13].

The analysis by intensity and domain indicated unique results. Students self-reported 4166.250 (3676.714) MET total MVPA, 2552.736 (2388.239) MET recreational activity and 2219.115 (3351.094) MET work but only 2593.650 (1052.028) min/week sedentary time. These results could be explained with the specific lifestyle of these university students. Participants were asked to take their studies as work into consideration and their majors were physiotherapy and recreation in high proportion in the sample. They exercise more and should be more physically active during their studies (practical lessons) and are also more interested in healthy lifestyle than other students. However, as young adults they made minimal efforts regarding housework as described with 626.098 (1003.992) total domestic MET.

Relatively high total walking 2018.479 (2024.153) MET and on the contrary relative low active transportation 1755.993 (1393.393) MET was found, which could be explained with the structure of the campus. It has a fragmented structure with more separated buildings in different places in the city; these are too close for cycling, moreover, the characteristics of the city do not support cycling as a mode of transport either.

However, Dinger et al. also studied college students (age 20.8 ± 1.5 years), and found significantly lower results in general with IPAQ-SF; these students spent on average only 124.6 min/week (females 94.8, males 209.4) with vigorous and 108.5 min/week (females 98.7, males 136.1) with moderate PA. If we convert our result, Hungarian students spent 257.1 min (females 201.9, males 325.7) with vigorous and 348.9 with moderate activity weekly. Different activity patterns were found with accelerometers as well; comparing averages of US to HU students 39.9 vs 6.5 (females 38.5 vs 3.9, males 43.4 vs 9.5) vigorous and 245.0 vs. 338.6 (females 224.0 vs 343.5, males 303,8 vs 332.9) moderate activity were registered. US students performed more vigorous but less moderate activity than HU students [30].

Boon and co-authors presented their findings with respect to age and found similar activity patterns in the group of young adults (18–35 years, mean 22.3 ± 4.2 years). Accumulated results over 7 days (min/week) as measured by ActiGraph MTI accelerometer and IPAQ-L were total MVPA 330 ± 327 vs 1086 ± 1318, vigorous PA 41 ± 79 vs 645 ± 813 and moderate PA 289 ± 292 vs 441 ± 808 respectively. A robust gender difference was found, females spent significantly less time on PA in total and also regarding all intensity PA – as measured with both methods [31]. We only found significant differences between genders according to the ActiGraph (*p* = 0.048) and IPAQ-HL vigorous activities (*p* = 0.017).

In a Spanish population study with reference to concurrent validity Roman-Vinas et al. found with MTI uni-axial accelerometers similar, perhaps a shade weaker correlation for total PA (*r* = 0.29), vigorous PA (*r* = 0.79) and moderate PA (*r* = 0.15). In this study multiple subjective measures were also found. Comparing the accelerometer and IPAQ data, they reported 16.769-times higher vigorous activity and 4.113-times higher moderate activity in questionnaire compared to objective results [32].

The Brazilian version of IPAQ-L largely overestimated self-reported PA results as well [33]. In this stratified random representative sample (*n* = 1572), but without direct measures, male and female respondents also showed unusually high levels of PA in household and work-related dimensions. They registered very high values overall, 83% of males and 89% of females reached the 150 min per week MVPA limit, calling into question the exclusive use of the Brazilian version of IPAQ-L for community health strategies [33].

Cleland et al. published contradictory results regarding older adults (age 71.8 ± 6.6 years) in the United Kingdom, they underestimated their level of MVPA and sitting time when completing IPAQ-L. However, accelerometer (Actigraph GT3X+) data was only 1.337-times more by MVPA, 1.623-times more by sedentary behaviour on week days and 1.671 by SB on weekend days than self-reported data; an error arose by Bland-Altman analysis on weekdays and on weekend days as well [34]. Hagströmer et al. also reported lower HEPA (the same like MVPA) and moderate PA scores with IPAQ than with MTI activity monitors (7.4 ± 9.5 vs 10.8 ± 3.4 and 5.1 ± 6.9 vs 9.1 ± 2.7 h/week respectively) on a modest Swedish sample (*n* = 42) [12]. Subsequently, comparing subjective and objective measures of PA in a population sample (*n* = 980) higher vigorous PA was found with MTI, but moderate accelerometer results remain lower than moderate plus walking activity by IPAQ-L [35].

However, some overestimation in self-report results is more common, Lee et al. describe three studies [30, 36, 37] using IPAQ-SF and accelerometer data with Freedson cut-off in a systematic review, which over-report with 101–173% MET-min/week compared to objective criterion on US, Chinese and Australian samples [38]. During the validation study of the long form of the questionnaire in New Zealand 165% overestimation of total PA was also described [31].

In our study the discrepancy of subjective and objective measures did not reach the critical limit and the following explanation can be provided. Despite the fact that there was no significant correlation between vigorous intensity activities, the vigorous activity of IPAQ-HL was significantly correlated with moderate accelerometer data (*R* = 0.507, *p* < 0.001) and total MVPA accelerometer data (*R* = 0.483, *p* < 0.001). It can be hypothesised, that respondents perceived and self-reported their activity as more intensive, and rated it as vigorous but the final result became balanced with MVPA.

The aim of the development of IPAQ was to assess suitable, internationally comparable measures of PA on population levels across countries. Therefore, we also compared our data with results from similar but also with different socio-cultural regions [39]. A former study on gender differences was conducted in relation to health status and physical activity habits of Czech, Polish, Hungarian and Slovak university students (*n* = 2237, age 19.497 ± 2.948 years). However, Visegrad countries are not in the vanguard regarding PA, only 21–35% of the general population exercises weekly, students provided more favourable data: 43.8% of female and 57.3% of male students were classified as highly active. Using IPAQ-L significant differences were revealed in every domain of activity (*p* < 0.001), the population of Polish students showed gender differences (*p* < 0.001) regarding vigorous and moderate activity and walking and Slovak and Hungarian students’ total MET/week and walking activities differ significantly regarding genders since women seemed to be more active in walking in their everyday lives (*p* < 0.001) [40].

In the original study of the IPAQ Committee results on repeatability were very good, around 0.8. Our data also showed very good reproducibility with Sperman’s rank correlation coefficients ranging from 0.765 (total work) to 0.985 (total vigorous) [13]. We also compered our result from a more methodological point of view with different socio-cultural regions. Studies showed mostly heterogeneous results regarding ICC. Studied a group of public university employees (*n* = 81) working in Malaysia - ICC scores of IPAQ-M showed moderate to good correlations (ICC = 0.54–0.92; *p* < 0.001) by intensity and domains [41]. Cultural adaptation of IPAQ-L in Nigeria among adult population revealed similarly good evidence of test-retest reliability with > 0.75 ICC except domestic PA (ICC = 0.38), limiting the validity of context specific PA behaviours [42]. Arabic version also showed a test-retest reliability > 0.70 ICC regarding overall score [29].

The present study is limited due to moderate sample size and convenient recruitment method; participants who already prefer a physically active lifestyle were more interested and more likely to be in the sample. We should also expand the range of limitations with a geographical, educational and age-related aspect, as the participation was limited to the University of Pécs.

## Conclusions

Our results indicate acceptable criterion validity for total physical activity, moderate to vigorous physical activity and moderate physical activity, moderate validity for vigorous activity and good reproducibility for vigorous moderate to vigorous and moderate activities for the Hungarian version of the long form of the International Physical Activity Questionnaire in the studied population. Nevertheless, like analogous self-reports in other languages, it overestimates the time spent on physical activity. In conclusion IPAQ-HL proved to be a reasonably valid measure for population prevalence epidemiological studies and is recommended for use to develop public health policy recommendations or to optimize public health interventions.

## Data Availability

The dataset supporting the conclusions of this article is available from the corresponding author on reasonable request.

## Abbreviations

BMI: Body mass index
CA: Cronbach’s Alpha
CEE: Central and Eastern European
EU: European Union
HEPA: Health-enhancing physical activity
ICC: Intraclass correlation coefficient
IPAQ: International Physical Activity Questionnaire
IQR: Interquartile range
KMO: Kaiser–Meyer–Olkin
MET: Metabolic equivalent of task
MVPA: Moderate to vigorous physical activity
PAQs: Physical activity questionnaires
PCA: Principal component analysis
SB: Sedentary behaviour
SD: Standard deviation
WHO: World Health Organization
